# Ultrasensitivity and bistability in covalent-modification cycles with positive autoregulation

**DOI:** 10.1098/rspa.2021.0069

**Published:** 2021-08

**Authors:** Cailan Jeynes-Smith, Robyn P. Araujo

**Affiliations:** ^1^ School of Mathematical Sciences, Queensland University of Technology, Brisbane, Australia; ^2^ Institute of Health and Biomedical Innovation (IHBI), Brisbane, Australia

**Keywords:** chemical reaction networks, enzymes, post-translational modifications, reaction kinetics, positive autoregulation

## Abstract

Switch-like behaviours in biochemical networks are of fundamental significance in biological signal processing, and exist as two distinct types: ultra-sensitivity and bistability. Here we propose two new models of a reversible covalent-modification cycle with positive autoregulation (PAR), a motif structure that is thought to be capable of both ultrasensitivity and bistability in different parameter regimes. These new models appeal to a modelling framework that we call *complex-complete*, which accounts fully for the molecular complexities of the underlying signalling mechanisms. Each of the two new models encodes a specific molecular mechanism for PAR. We demonstrate that the modelling simplifications for PAR models that have been used in previous work, which rely on Michaelian approximations, are unable to accurately recapitulate the qualitative signalling responses supported by our detailed models. Strikingly, we show that complex-complete PAR models are capable of new qualitative responses such as one-way switches and a ‘prozone’ effect, depending on the specific PAR-encoding mechanism, which are not supported by Michaelian simplifications. Our results highlight the critical importance of accurately representing the molecular details of biochemical signalling mechanisms, and strongly suggest that the Michaelian approximation is inadequate for predictive models of enzyme-mediated chemical reactions with added regulations such as PAR.

## 1. Introduction

**Figure 1.**
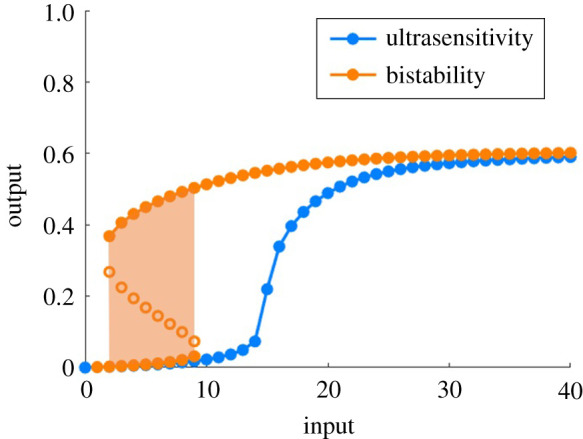
Distinctive qualitative features of ultrasensitive and bistable dose–response profiles. Ultrasensitivity (blue curve) corresponds to a monostable, yet steeply sigmoidal, steady state dose–response profile. By contrast, bistability (orange curve) arises when two stable steady states (separated by an unstable steady state) exist for some range of inputs (indicated by the shaded orange region). (Online version in colour.)

The capacity for collections of biochemical reactions to respond in a switch-like, or *all-or-none*, manner is of fundamental significance in biological signal processing, and has been widely observed in many different signalling contexts [1–3]. Biochemical switch-like responses can exist as two distinct types [4,5]: ultrasensitivity, which is a steeply sigmoidal, monostable, dose–response profile; and bistability, a commonly observed form of multistationarity, which constrains the system to exist in one of two possible stable steady states for a given level of stimulus (figure 1).

Ultrasensitivity has been implicated in functionally important switching mechanisms across a wide variety of biological contexts, ranging from cell signalling pathways [6,7], budding in yeast [8,9], maturation of xenopus oocytes [10,11] and embryonic differentiation [12]. More recently, ultrasensitivity has been recognized as an essential component in many robust perfect adaptation (RPA) mechanisms [13–15]. RPA is the ability of a system to asymptotically track a fixed ‘set-point’ following persistent perturbations to its interacting elements. This keystone signalling response is thought to be an essential characteristic of all evolvable and self-regulating biosystems [14,15], and has been ubiquitously observed across all levels of biological organization, from chemotaxis in single-celled organisms [16–27], to complex sensory systems [28–38]. Importantly, the dysregulation of RPA is believed to be linked to disorders such as cancer progression, drug addiction, chronic pain and metabolic syndrome [39–44].

While the history of mathematical approaches to the study of ultrasensitivity-generating mechanisms may be traced back to the work of AV Hill [45] on cooperative binding in haemoglobin, the seminal work of Goldbeter & Koshland [46] on *zero-order* ultrasensitivity has exerted a profound influence on our modern understanding of ultrasensitivity. Indeed, it was Goldbeter & Koshland [46] who formally defined an ultrasensitive response as ‘an output response that is more sensitive to change in stimulus than the hyperbolic (Michaelis–Menten) equation’. Their landmark findings demonstrated that enzyme-mediated covalent-modification cycles (such as phosphorylation/dephosphorylation cycles, or methylation/demethylation cycles, for instance) are capable of generating sensitivities comparable with cooperative enzymes with high Hill coefficients when the interconverting enzymes operate in their ‘zero-order’ regions (i.e. in the region of saturation with respect to their protein substrates). Importantly, this was the first known example of *unlimited* ultrasensitivity: unlike the response of cooperative enzymes, where the steepness of the dose–response profile was limited, the zero-order mechanism allowed steepness to be increased indefinitely by ‘tuning’ certain parameter groups. More recently, a number of additional ultrasensitivity-generating mechanisms have been identified [47,48], including inhibitor-generated ultrasensivity and substrate competition [48–51], cooperative binding [52,53], positive feedback [3,6,54], multisite phosphorylation [9,12,55] and negative feedback [8,48]. Some mathematical models also support the idea that cascade structures can further amplify ultrasensitivity if some degree of ultrasensitivity already exists at the level of individual tiers in the cascade [11]. The extent to which this latter factor might contribute to ultrasensitivity in large-scale signalling networks, in signal transduction and metabolism for instance, is still poorly understood [56–58].

In contrast to ultrasensitivity, bistability gives rise to a discontinuous switching mechanism. Bistability is widely believed to play a vital functional role in gene regulatory networks [59–63], cell differentiation [64,65], cell-cycle regulation [66,67], lineage commitment during development [2], exit from quiescence in mammalian cells [68] and biochemical and working memory [69,70]. Moreover, a bistable switch can exist as a one-way (irreversible) switch, or a two-way (toggle) switch. Toggle switches are reversible but exhibit hysteresis, whereby once the switch has been ‘tripped’ by a suitable increase in stimulus, propelling the system to its upper steady state, a *much larger decrease* in input stimulus is required to bring the system back down to its lower steady state, a functionally important response in the context of fluctuating inputs [2,69,71]. One-way switches, on the other hand, can never return the system to its lower steady state once the switch has been tripped. The aberrant formation of such one-way switches is thought to play a particularly pernicious role in cancer signalling dysregulation, by driving the constitutive activation of oncoproteins [5,72,73]. Mathematically, bistability is thought to arise from a variety of underlying mechanisms, including: positive autoregulation (PAR) and positive feedback, where a molecule either directly or indirectly promotes its own creation [54,59,60,62,68,69,74,75]; cooperative binding, where the binding of one molecule enhances the binding of subsequent molecules [66,76]; antagonism, where one molecule benefits at the loss of another [65,77]; and double negative feedback, a cycle in which two interacting molecules mutually inhibit each other [54].

Although emergent network behaviours such as bistability and ultrasensitivity, and the related phenomenon of RPA, have been the focus of many mathematical models, the detailed molecular mechanisms that might support these functions in ‘real’, highly complex signalling interactions are yet to be understood. Indeed, a frontier research problem in biochemical signalling is the question of how specialized signal processing functions such as ultrasensitivity are implemented at the *microscale* of complex networks, through the highly intricate interactions of collections of proteins [15]. Mathematical models of biochemical signal processing to date have typically been highly simplified—either by neglecting many of the proteins known to be involved, and frequently by simplifying the mathematical nature of their interactions—most notably through the (often indiscriminate) use of the Michaelis–Menten equation, involving the quasi-steady-state assumption (QSSA) on intermediate protein–protein complexes.

The Michaelis–Menten rate law has a long history in the mathematical modelling of enzyme–substrate interactions, having originally been proposed through the seminal studies of Henri [78], Michaelis & Menten [79] and Briggs & Haldane [80] in the early decades of the twentieth century. Its limitations to the study of even simple enzyme–substrate interactions are now well known [81,82], and a variety of alternative theoretical methods, mostly employing alternative QSSAs, have been developed to yield modelling simplifications that are applicable to a broader range of parameter regimes than the standard Michaelis–Menten equation, with its ‘standard’ QSSA (see [81] for a detailed review). Nevertheless, the more complicated enzyme–substrate interactions that readily arise in biological networks, involving multisite chemical modifications, positive or negative autoregulation of substrate molecules, and other molecular intricacies, are currently beyond the reach of most modelling simplifications involving alternative QSSAs [14,15]. Indeed, even small such collections of relatively simple enzyme-mediated reactions can involve the formation of a number of transient, intermediate molecular states and protein–protein complexes, and can quickly give rise to complicated mathematical descriptions when all such states are explicitly accounted for. Such intricate enzyme–substrate interactions often continue to be modelled using Michaelian approximations [13,15], although the mathematical consequences of doing so have not been examined in detail to date to our knowledge.

In the present paper, we consider the mathematical modelling of covalent-modification cycles with a set of added regulations known as *PAR*. We are motivated in large part by the influential RPA study by Ma *et al.* [13], which employs Michaelian models of PAR to suggest that positively autoregulated covalent-modification cycles are supportive of ultrasensitivity, and hence RPA, in very specific parameter regimes. But to what extent might ultrasensitivity, and ultimately RPA, truly prevail if the detailed intermolecular mechanisms of PAR, when all associated intermediate protein–protein complexes, are explicitly accounted for in the model? Are the Michaelian models able to fully recapitulate the qualitative responses of more detailed models that account explicitly for molecular mechanisms? And is the relationship between qualitative response and parameter regime reliable in the Michaelian framework?

We attempt to offer important new insights into these questions by proposing several new, simple, yet detailed, mathematical models of covalent-modification cycles incorporating PAR. We appeal to a mathematical framework we call *complex-complete* wherein the molecular details of each specific PAR mechanism will be considered explicitly, and all intermediate protein–protein complexes accounted for. We will consider the capacity of each such model to exhibit bistability and ultrasensitivity, through model simulations over a wide range of parameters, and compare these outcomes with the behaviour supported by the well-established Michaelian model of PAR [13].

**Figure 2.**
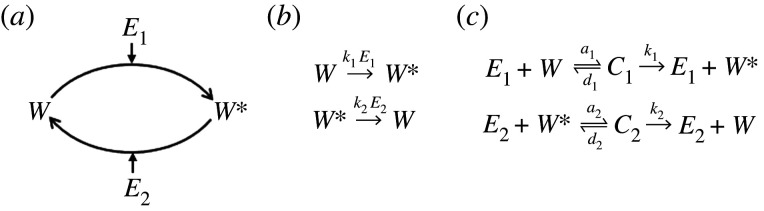
A mathematical framework for the modelling of a single covalent-modification cycle. (*a*) A schematic of a reversible covalent-modification cycle in which a protein substrate (*W*) is chemically modified by an enzyme, *E*_1_, into an active form (W*). The active form is, in turn, converted back to its unmodified form through the action of a second, independent, enzyme, *E*_2_. (*b*) A ‘complex-free’ mechanism, in which enzyme concentrations are incorporated into the biochemical rate constants ($k_{1},k_{2}$), can be used as the basis of a simplified mass-action model of the cycle. (*c*) A reaction mechanism that explicitly includes transient enzyme-substrate complexes ($C_{1},C_{2}$) can be used as the basis of either a *complex-complete* mass action model, or a Michaelian model.

As a backdrop to these novel complex-complete models of PAR, we first briefly review the two main classes of simplified frameworks for the mathematical modelling of covalent-modification cycles: simplified mass-action models and Michaelian models (figure 2).

### (a) Modelling frameworks for covalent-modification cycles

Consider the basic structure of a covalent-modification cycle (figure 2*a*), where an enzyme, *E*_1_, binds to a substrate, *W*, and converts it to a chemically modified form, W*(*t*). A second enzyme, *E*_2_, can then bind to the chemically modified protein, W*, and convert it back to its original, unmodified, form, *W*.

Simplified mass-action models exclude intermediate protein–protein complexes (figure 2*b*). A simplified mass-action model of a covalent-modification cycle thus produces a single ordinary differential equation (ODE) for the concentration of the output, W*(*t*), as in equation (1.1),

1.1 $$\frac{\text{d}W^{*}}{\text{d}t}=k_{1}E_{1}(W_{\text{tot}}-W^{*})-k_{2}E_{2}W^{*},$$

with

1.2 $$W_{\text{tot}}=W+W^{*},$$

as a *conservation relation*, where *W*_tot_, assumed fixed, denotes the total concentration of substrate protein present in either form. Thus, equation (1.2) yields the concentration of the unmodified protein, *W*(*t*), once W*(*t*) has been obtained from equation (1.1).

By contrast, Michaelis–Menten kinetics do take into account the existence of intermediate complexes in the system (figure 2*c*), but their explicit consideration is avoided through the application of the QSSA. By the QSSA, complex association (*a*_*i*_) and dissociation (*d*_*i*_) processes are assumed to occur on a significantly faster timescale than the catalysis (*k*_*i*_) reaction. Moreover, the steady-state concentrations of the complexes (there being two enzyme-substrate complexes, *C*_1_ and *C*_2_, in a single covalent-modification cycle), having rapidly been reached, are assumed to be negligible in comparison with the total protein concentration (i.e. $W_{\text{tot}}=W^{*}+W+C_{1}+C_{2}\approx W^{*}+W$). We thereby obtain a single equation for W*(*t*) (equation (1.3)), which features parameter groups known as Michaelis constants, $K_{1}=(d_{1}+k_{1})/a_{1}$ and $K_{2}=(d_{2}+k_{2})/a_{2}$,

1.3 $$\frac{\text{d}W^{*}}{\text{d}t}=\frac{k_{1}E_{1\text{tot}}(W_{\text{tot}}-W^{*})}{K_{1}+W_{\text{tot}}-W^{*}}-\frac{k_{2}E_{2\text{tot}}W^{*}}{K_{2}+W^{*}}.$$

To equation (1.3), we can add the following three conservation relations:

1.4 $$\begin{array}{rl} & W_{\text{tot}}\approx W+W^{*},\\ & E_{1\text{tot}}=E_{1}+C_{1}\approx E_{1}\\ \;\text{and}\; & E_{2\text{tot}}=E_{2}+C_{2}\approx E_{2}. \end{array}\}$$

The first of these determines the concentration of *W*(*t*) once W*(*t*) has been established from (1.3), assuming the total protein abundance, *W*_tot_, to be a constant. Because the two intermediate complexes, *C*_1_ and *C*_2_, are assumed to be of negligible concentration in the Michaelian framework, the total enzyme concentrations, *E*_1tot_ and *E*_2tot_ (also assumed to be constants), are approximated by the concentrations of the corresponding free enzymes, *E*_1_ and *E*_2_, as indicated in the final two conservation relations in (1.4).

In contradistinction to the two simplification strategies noted above, we refer to models that consider all signalling events and intermediate molecules explicitly, thereby providing a detailed representation of the signalling mechanisms, as *complex-complete mass-action models*. The complex-complete mass-action model ‘induced’ by the chemical reaction network in figure 2*c* can be expressed as a system of four ODEs (1.5) and three conservation equations (1.6),

1.5 $$\begin{array}{rl} & \frac{\text{d}W}{\text{d}t}=d_{1}C_{1}+k_{2}C_{2}-a_{1}WE_{1},\\ & \frac{\text{d}W^{*}}{\text{d}t}=d_{2}C_{2}+k_{1}C_{1}-a_{2}W^{*}E_{2},\\ & \frac{\text{d}C_{1}}{\text{d}t}=a_{1}WE_{1}-d_{1}C_{1}-k_{1}C_{1},\\ & \frac{\text{d}C_{2}}{\text{d}t}=a_{2}W^{*}E_{2}-d_{2}C_{2}-k_{2}C_{2} \end{array}\}$$

and

1.6 $$\begin{array}{rl} & W_{\text{tot}}=W+W^{*}+C_{1}+C_{2},\\ & E_{1\text{tot}}=E_{1}+C_{1},\\ & E_{2\text{tot}}=E_{2}+C_{2}. \end{array}\}$$

A detailed description of the process by which polynomial dynamical systems are induced by chemical reaction networks, under mass-action kinetics, is given in [83].

As mathematical models grow in size and complexity, simplified mass-action and Michaelian descriptions of the various components of the system can significantly reduce the number of variables and parameters to be considered. On the other hand, these benefits come at the expense of neglecting signalling events which could potentially alter the qualitative nature of the system’s response in significant ways [15,77,84]. Importantly, Goldbeter & Koshland’s seminal work on zero-order ultrasensitivity [46] has suggested that the Michaelian model of a covalent-modification cycle, equation (1.3), provides a good approximation to the ultrasensitive behaviour of the complex-complete mass-action model *provided* that $E_{1\text{tot}},E_{2\text{tot}}\ll W_{\text{tot}}$, a parametric condition unlikely to obtain in many signal transduction networks, or other highly complex bionetworks in nature [14]. Moreover, if additional regulations such as autoregulatory interactions are present in the system, with an inevitable increase in the number of intermediate protein–protein complexes, it is unclear if simplified Michaelian descriptions of the system would be capable of recapitulating the qualitative behaviours of the corresponding complex-complete models for *any* parameter regime.

Although the larger number of variables and parameters often necessitates numerical, rather than analytical, approaches, these models allow the complexity of the system to speak for itself, and can be used to verify the extent to which simplified models of signalling motifs can recapitulate the behaviours of more detailed, accurate and mechanistic mathematical descriptions.

We now proceed to compare three different models of a covalent-modification cycle with PAR—the well-established Michaelian model studied by Ma *et al*. [13], along with two novel complex-complete mass-action models.

### (b) A Michaelian model of positive autoregulation

In order to generate ultrasensitivity (and hence RPA when suitably embedded into an appropriate network topology) via PAR of a covalent-modification cycle, Ma *et al*. [13] used an equation of the following form:

1.7 $$\frac{\text{d}W^{*}}{\text{d}t}=\frac{k_{1}E_{1}W^{*}(W_{\text{tot}}-W^{*})}{K_{1}+(W_{\text{tot}}-W^{*})}-\frac{k_{2}E_{2}W^{*}}{K_{2}+W^{*}}.$$

We refer to this model hereafter as PAR-MM. Here it is assumed that the rate of increase of W* occurs in direct proportion to $E_{1}W^{*}$ (rather than just *E*_1_, as is the case for the Michaelian model without added regulations), thereby enabling the output molecule to enhance its own production. It is clear from the form of equation (1.7) that if $K_{1}\ll W_{\text{tot}}-W^{*}$ and $K_{2}\gg W^{*}$, then at steady state, $0\approx W^{*}(k_{1}E_{1}-k_{2}E_{2}/K_{2})$. Thus, the system can exhibit unlimited ultrasensitivity as this parametric limit is approached, with a near-vertical dose–response occurring at $E_{1\text{tot}}\approx E_{1}=(k_{2}/k_{1}K_{2})E_{2}$.

**Figure 3.**
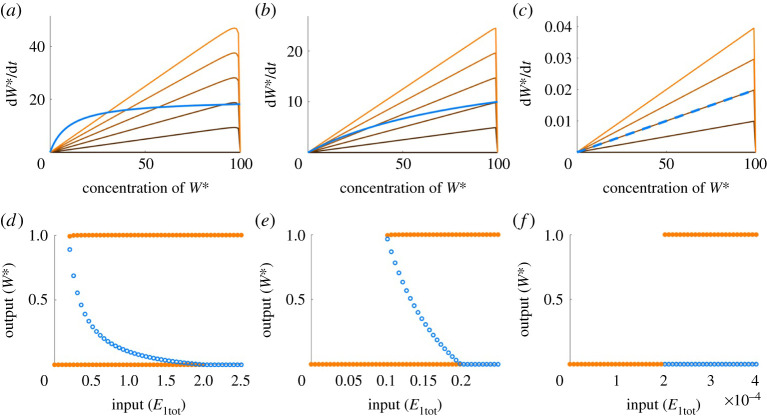
Bistability and ultrasensitivity in a Michaelian model of PAR. Figures (*a*–*c*) plot the positive and negative components of the rate of change in W* from equation (1.7), for three different parameter sets. In each case, the negative component is represented by the blue line. The positive component is represented by the solid line that changes from black to orange with increasing value of *E*_1_. The intersection of the positive and negative components indicates the steady states of the system, plotted in (*d*–*f* ), respectively. Stable steady states are represented by solid circles, unstable states represented by open circles. Parameters: (all plots) $k_{1}=k_{2}=1$, $W_{\text{tot}}=100$, $E_{2}=20$. (*a*,*d*): $K_{1}=10^{-1}$ and $K_{2}=10$; (*b*,*e*): $K_{1}=10^{-2}$ and $K_{2}=10^{2}$; (*c*,*f* ): $K_{1}=10^{-5}$ and $K_{2}=10^{5}$. (Online version in colour.)

In figure 3, we examine how the Michaelis constants of PAR-MM are the key drivers of both bistability and (unlimited) ultrasensitivity in this model. In particular, for an initial choice of $K_{1}=10^{-1}$ and $K_{2}=10$ (figure 3*a*,*d*), the rate of increase in W* (orange/black lines), as given by the first term in equation (1.7), intersects the rate of decrease (blue line) in multiple places, indicating the existence of multiple steady states (bistability, in this case) for some values of *E*_1_. As we increase the value of *K*_2_ and decrease *K*_1_, we observe a narrowing in the range of values of *E*_1_ for which bistability obtains (figure 3*b*,*e*). This occurs as the negative rate of change approaches the same form as the positive rate of change. For $K_{1}\ll W_{\text{tot}}-W^{*}$ and $K_{2}\gg W^{*}$, the two curves approach an exact match, and complete overlap, for $E_{1}=(k_{2}/k_{1}K_{2})E_{2}$ (figure 3*c*,*f* ). Under these conditions, every non-zero value of W* is compatible with a steady state at $E_{1}=(k_{2}/k_{1}K_{2})E_{2}$, a scenario that corresponds to unlimited ultrasensivity (i.e. a vertical dose–response curve at the noted value of *E*_1_). Note that in the Michaelian model of PAR by Ma *et al.* [13], being identical in form to PAR-MM, there is no consideration of *how* the output molecule W* might upregulate its own production through its interactions with other molecules, and thus no accounting for any particular autoregulatory *mechanism*. As a consequence, there is no way to determine how well this model might reflect PAR mechanisms in ‘real’ biochemical reaction networks. In order to consider the role of the *mechanism* in PAR, we propose two possible chemical reaction mechanisms: a direct positive autoregulatory mechanism (hereafter, PAR-d), and an indirect mechanism (hereafter, PAR-i).

### (c) A novel model of direct positive autoregulation

**Figure 4.**
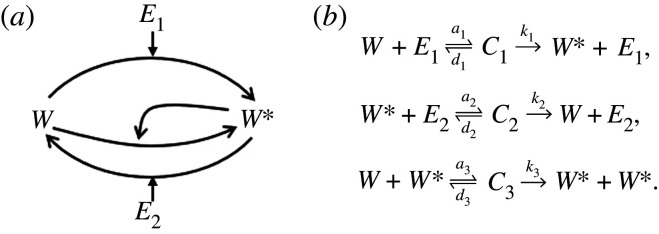
PAR-d System. (*a*) Schematic of the PAR-d mechanism. (*b*) Chemical reaction network corresponding to the PAR-d mechanism, comprising three groups of reactions: (i) *E*_1_ binding reversibly to *W*, forming a complex *C*_1_, and catalysing the production of W* from *W*; (ii) *E*_2_ binding reversibly to W*, forming a complex *C*_2_, and catalysing the production of *W* from W*; and (iii) W* binding reversibly to *W*, forming a complex *C*_3_, and catalysing the conversion of *W* to W*.

In order to implement a direct PAR mechanism, we supplement the basic covalent-modification cycle (figure 2*c*) to include a third set of reactions whereby the output, W*, binds *in trans* to the unmodified protein *W*, thereby catalysing the conversion of the latter to W*. In the process of doing so, an intermediate protein–protein complex comprising both *W* and W* is formed. We represent this process schematically in figure 4*a* and by the chemical reaction network in figure 4*b*.

This chemical reaction network, through the law of mass-action, induces the following system of ordinary differential equations:

1.8 $$\begin{array}{rl} & \frac{\text{d}W}{\text{d}t}=d_{1}C_{1}+d_{3}C_{3}+k_{2}C_{2}-a_{1}WE_{1}-a_{3}WW^{*}, \end{array}$$

1.9 $$\begin{array}{rl} & \frac{\text{d}W^{*}}{\text{d}t}=d_{2}C_{2}+d_{3}C_{3}+k_{1}C_{1}+2k_{3}C_{3}-a_{2}W^{*}E_{2}-a_{3}WW^{*}, \end{array}$$

1.10 $$\begin{array}{rl} & \frac{\text{d}E_{1}}{\text{d}t}=d_{1}C_{1}+k_{1}C_{1}-a_{1}WE_{1}, \end{array}$$

1.11 $$\begin{array}{rl} & \frac{\text{d}E_{2}}{\text{d}t}=d_{2}C_{2}+k_{2}C_{2}-a_{2}W^{*}E_{2}, \end{array}$$

1.12 $$\begin{array}{rl} & \frac{\text{d}C_{1}}{\text{d}t}=a_{1}WE_{1}-d_{1}C_{1}-k_{1}C_{1}, \end{array}$$

1.13 $$\begin{array}{rl} & \frac{\text{d}C_{2}}{\text{d}t}=a_{2}W^{*}E_{2}-d_{2}C_{2}-k_{2}C_{2} \end{array}$$

1.14 $$\begin{array}{r} \;\text{and}\;\frac{\text{d}C_{3}}{\text{d}t}=a_{3}WW^{*}-d_{3}C_{3}-k_{3}C_{3}. \end{array}$$

The summation of equations (1.8) through (1.14) yields a conservation equation for the substrate protein,

$$W_{\text{tot}}=W+W^{*}+C_{1}+C_{2}+2C_{3}.$$

Likewise, summation of equations (1.10), and (1.12), and of equations (1.11), and (1.13), yield conservation equations for the two enzymes:

$$E_{1\text{tot}}=E_{1}+C_{1}$$

and

$$E_{2\text{tot}}=E_{2}+C_{2}.$$

### (d) A novel model of indirect positive autoregulation

**Figure 5.**
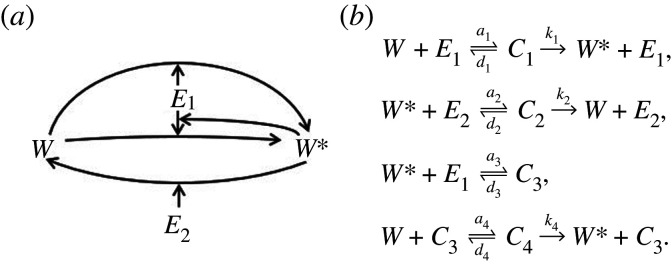
PAR-i System. (*a*) Schematic of the PAR-i mechanism. (*b*) A chemical reaction network for PAR-i. This includes four reaction groups: (i) *E*_1_ binding reversibly to *W*, forming a complex *C*_1_, and catalysing the production of W* from *W*; (ii) *E*_2_ binding reversibly to W* forming a complex *C*_2_, and catalysing the production of *W* from W*; (iii) W* binding reversibly with *E*_1_ to form a complex *C*_3_; and (iv) *C*_3_ (containing enzyme *E*_1_) binding reversibly with *W*, forming a complex *C*_4_, catalysing the conversion of *W* to W*.

For the indirect implementation of PAR, we introduce two new reaction groups to the reversible covalent-modification cycle. First, we add a reaction in which the output, W*, can bind reversibly with the enzyme, *E*_1_, forming the transient complex *C*_3_. Then, in a second reaction group, the complex *C*_3_ can bind reversibly to *W*, forming another complex *C*_4_, which catalyses the conversion of *W* to W*. In this way, the binding of W* to *E*_1_ (producing *C*_3_) alters the efficiency of the enzyme *E*_1_ through an allosteric interaction. Thus, in contrast to PAR-d, where W* catalyses its own production from the unmodified form *W*, W* now enhances its own production through an indirect mechanism, through its interactions with *E*_1_. We illustrate the PAR-i mechanism by the schematic in figure 5*a*, and produce a corresponding chemical reaction network in figure 5*b*.

By the law of mass-action, we obtain a system of ordinary differential equations from the chemical reaction network in figure 5*b*:

1.15 $$\begin{array}{rl} & \frac{\text{d}W}{\text{d}t}=d_{1}C_{1}+k_{2}C_{2}+d_{4}C_{4}-a_{1}WE_{1}-a_{4}WC_{3}, \end{array}$$

1.16 $$\begin{array}{rl} & \frac{\text{d}W^{*}}{\text{d}t}=k_{1}C_{1}+d_{2}C_{2}+d_{3}C_{3}+k_{4}C_{4}-a_{2}W^{*}E_{2}-a_{3}W^{*}E_{1}, \end{array}$$

1.17 $$\begin{array}{rl} & \frac{\text{d}E_{1}}{\text{d}t}=d_{1}C_{1}+k_{1}C_{1}+d_{3}C_{3}-a_{1}WE_{1}-a_{3}W^{*}E_{1}, \end{array}$$

1.18 $$\begin{array}{rl} & \frac{\text{d}E_{2}}{\text{d}t}=d_{2}C_{2}+k_{2}C_{2}-a_{2}W^{*}E_{2}, \end{array}$$

1.19 $$\begin{array}{rl} & \frac{\text{d}C_{1}}{\text{d}t}=a_{1}WE_{1}-d_{1}C_{1}-k_{1}C_{1}, \end{array}$$

1.20 $$\begin{array}{rl} & \frac{\text{d}C_{2}}{\text{d}t}=a_{2}W^{*}E_{2}-d_{2}C_{2}-k_{2}C_{2}, \end{array}$$

1.21 $$\begin{array}{rl} & \frac{\text{d}C_{3}}{\text{d}t}=a_{3}W^{*}E_{1}+d_{4}C_{4}+k_{4}C_{4}-d_{3}C_{3}-a_{4}WC_{3} \end{array}$$

1.22 $$\begin{array}{r} \;\text{and}\;\frac{\text{d}C_{4}}{\text{d}t}=a_{4}WC_{3}-d_{4}C_{4}-k_{4}C_{4}, \end{array}$$

The summation of equations (1.15), (1.16), (1.19), (1.20), (1.21) and (1.22) gives a conservation equation for the substrate protein,

$$W_{\text{tot}}=W+W^{*}+C_{1}+C_{2}+C_{3}+2C_{4}.$$

Similarly, the summation of equations (1.17), (1.19), (1.21) and (1.22), and of equations (1.18) and (1.20), yield conservation equations for the two interconverting enzymes:

$$E_{1\text{tot}}=E_{1}+C_{1}+C_{3}+C_{4}$$

and

$$E_{2\text{tot}}=E_{2}+C_{2}.$$

## 2. Results

Our overarching goal is to determine the extent to which bistability (as a one-way switch and/or as a toggle switch) and ultrasensitivity are obtained in our complex-complete models of PAR (PAR-d and PAR-i), and to establish the similarities and differences in the predictions of these detailed models in comparison with the simpler Michaelian model of PAR (PAR-MM).

With this goal in mind, we commenced our study with a preliminary exploratory parameter search in which we classified the qualitative nature of the dose–response profiles for both the PAR-d and PAR-i motifs for approximately 1000 random parameter sets, each parameter taking values in the range ($10^{-6}$, $10^{6}$). It was striking to observe that ultrasensitive or bistable profiles were only obtained when $K_{1}/W_{\text{tot}},K_{2}/W_{\text{tot}}\ll 1$, that is, for parameter regimes corresponding to ultrasensitivity in the Goldbeter–Koshland model *without* PAR. Indeed, where $K_{1}/W_{\text{tot}}$ and $K_{2}/W_{\text{tot}}$ assumed larger values (say $K_{1}/W_{\text{tot}}$, $K_{2}/W_{\text{tot}}>1$), the dose–response profiles for both PAR-d and PAR-i models were invariably characterized by either low conversion to W* (i.e. a maximal steady state W* much lower than *W*_tot_) and/or a subsensitive dose–response profile.

**Figure 6.**
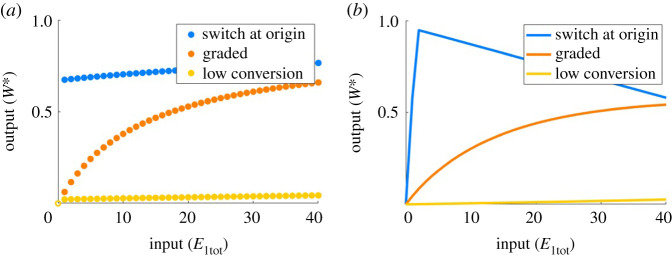
Representative dose–responses of PAR-d and PAR-i for $K_{1}/W_{\text{tot}}$, $K_{2}/W_{\text{tot}}>1$. Parameters: (all plots) $W_{\text{tot}}=100$, $E_{2\text{tot}}=20$. (*a*) switch at origin: $a_{1}=a_{2}=a_{3}=0.01$, $d_{1}=k_{1}=d_{2}=k_{2}=10$, $d_{3}=k_{3}=1$; graded: $a_{1}=a_{2}=0.1$, $d_{1}=k_{1}=d_{2}=k_{2}=d_{3}=k_{3}=10$, $a_{3}=0.01$; low conversion: $a_{1}=d_{1}=k_{1}=a_{2}=d_{2}=a_{3}=d_{3}=k_{3}=1$, $k_{2}=10$; (*b*) switch at origin: $a_{1}=a_{2}=0.01$, $d_{1}=k_{1}=d_{2}=a_{3}=10$, $k_{2}=d_{4}=k_{4}=1$, $d_{3}=0.1$, $a_{4}=100$; graded: $a_{1}=a_{2}=a_{4}=0.01$, $d_{1}=k_{1}=d_{2}=k_{2}=d_{3}=10$, $a_{3}=0.1$, $d_{4}=k_{4}=10$; low conversion: $a_{1}=d_{1}=k_{1}=a_{2}=d_{2}=a_{4}=d_{4}=k_{4}=1$, $k_{2}=d_{3}=10$, $a_{3}=0.1$. Solid circles indicate stable steady states; open circles indicate unstable steady states. (Online version in colour.)

As an illustrative example, we depict in figure 6 representative dose–response profiles for our PAR-d and PAR-i models using $K_{1}/W_{\text{tot}}$, $K_{2}/W_{\text{tot}}>1$, depicting typical subsensitive and low-conversion dose–response profiles that characterize this parameter regime. As shown, subsensitive responses were of two distinct types: gently sloping ‘graded’ responses, or a rapid conversion (switch) to maximal output at or near the origin, with weak dependence on input variations thereafter.

Moreover, our preliminary parameter searches made clear that the parameter choices that ‘drive’ the qualitative nature of the responses in the complex-complete models of PAR were vastly more complex and subtle than for PAR-MM. In this connection, we note that for PAR-MM the essential drivers of the model’s qualitative response are the Michaelis constants, *K*_1_ and *K*_2_. Indeed, by increasing *K*_2_ and decreasing *K*_1_, the model gives rise to a narrowing bistable region (figure 3*d*,*e*), and in the limit as $K_{2}\to \infty$ and $K_{1}\to 0$, unlimited ultrasensitivity obtains (figure 3*f* ). Thus, the qualitative nature of the system’s response is completely determined by the choice of *K*_1_ and *K*_2_.

**Figure 7.**
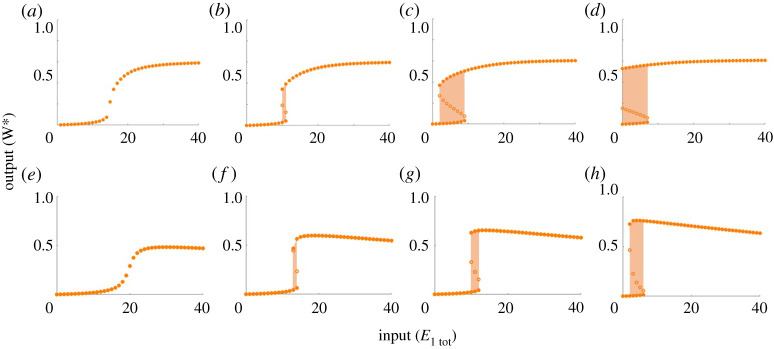
Representative collection of dose–responses of PAR-d and PAR-i for a single choice of Michaelis constants. Parameters: (all plots) $W_{\text{tot}}=100$, $E_{2\text{tot}}=20$, $a_{1}=d_{1}=k_{1}=a_{2}=d_{2}=k_{2}=1$, $K_{1}=K_{2}=2$, $K_{3}=200$. (*a*) $a_{3}=0.01$, $d_{3}=k_{3}=1$; (*b*) $a_{3}=0.02$, $d_{3}=k_{3}=2$; (*c*) $a_{3}=0.03$, $d_{3}=k_{3}=3$; (*d*) $a_{3}=0.04$, $d_{3}=k_{3}=4$; (*e*)–(*h*) $K_{4}=2$, $a_{3}=0.1$, $d_{3}=10$, $k_{3}=10$; (*e*) $a_{4}=d_{4}=k_{4}=1$; (*f* ) $a_{4}=d_{4}=k_{4}=3$; (*g*) $a_{4}=d_{4}=k_{4}=5$; (*h*) $a_{4}=d_{4}=k_{4}=30$. Bistable regions are highlighted by orange shading. Solid circles indicate stable steady states; open circles indicate unstable steady states. (Online version in colour.)

For the complex-complete models PAR-d and PAR-i, by contrast, it is intriguing to observe that the capacity for ultrasensitivity or bistability is *not* determined by the Michaelis constants alone, as we demonstrate in figure 7. In fact it is readily apparent that for both PAR-d (figure 7*a*–*d*) and PAR-i (figure 7*e*–*h*), a full range of behaviours including ultrasensitivity, subsensitivity (not shown) and bistability, may all be obtained with a single choice of Michaelis constants *K*_1_, *K*_2_ and *K*_3_ (as well as *K*_4_ in the case of PAR-i).

Having thus established a coarse-grained view of the requisite conditions for ultrasensitivity and bistability in our complex-complete models of PAR, we proceeded to examine the qualitative influence of each individual rate constant (*a*_*i*_, *d*_*i*_, *k*_*i*_), the total protein abundances (*W*_tot_ and *E*_2tot_, considered constants) as well as key parameter groups—the Michaelis constants (*K*_1_, *K*_2_, *K*_3_), and the dissociation constant (*K*_*d*_, appearing only in PAR-i)—under the parametric conditions $K_{1}/W_{\text{tot}}$, $K_{2}/W_{\text{tot}}\leq 0.1$, for both PAR-d and PAR-i. Each parameter, or parameter group, was then varied in turn over the range $\left(10^{-10},10^{10}\right)$ for a collection of fixed values for all other parameters. Linear stability analysis was then used to classify the stability of all steady states.

### (a) PAR-d exhibits both one-way and two-way switches, in addition to ultrasensitivity

Our extensive computational simulations reveal that the existence of bistability in the dose–response of PAR-d differs from that in PAR-MM in a number of fundamental ways. First, unlike PAR-MM, which only admits two-way (toggle) switches in its bistable regime, PAR-d also has the potential for one-way (irreversible) switches. In figure 7*a*–*d*, we see that altering a single parameter in the PAR-d model can shift its response from monostable ultrasensitivity, to a bistable two-way switch, through to a bistable one-way switch. Furthermore, as we noted above, the capacity for bistability in PAR-d is *not* determined by the Michaelis constants alone; in fact, for each of the three individual rate constants, *a*_*i*_, *d*_*i*_, *k*_*i*_, that comprise a Michaelis constant, *K*_*i*_, we discovered that it is the catalytic constant, *k*_*i*_, specifically, that controls the qualitative response of the system and the shape of its dose–response profile.

**Figure 8.**
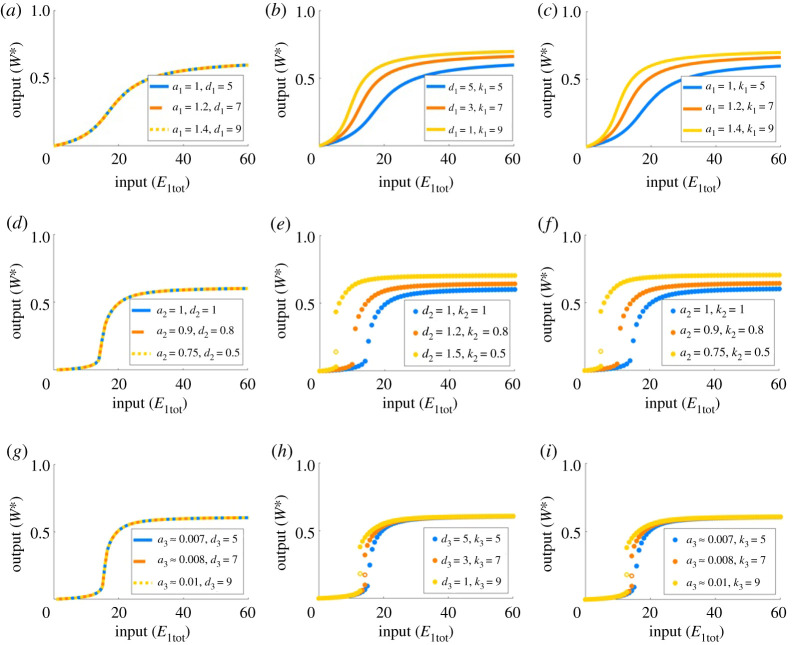
Varying rate constants in PAR-d. Pairs of independent rate constants are altered whilst holding the corresponding values of *K*_1_, *K*_2_ and *K*_3_ fixed. Parameters: $W_{\text{tot}}=100$ and $E_{2\text{tot}}=20$. (*a*)–(*c*): $a_{1}=a_{2}=d_{3}=k_{3}=1$, $d_{1}=k_{1}=d_{2}=k_{2}=5$, $a_{3}=0.01$; (*d*)–(*f* ): $a_{1}=d_{1}=k_{1}=a_{2}=d_{2}=k_{2}=d_{3}=k_{3}=1$, $a_{3}=0.01$; (*g*)–(*i*): $a_{1}=d_{1}=k_{1}=a_{2}=d_{2}=k_{2}=1$, $a_{3}=0.007$, $d_{3}=k_{3}=5$. Varied rate constants are identified in the figure legends. Solid circles indicate stable steady states; open circles indicate unstable steady states. (Online version in colour.)

We explore this finding further in figure 8, where we examine the various possible combinations of holding a Michaelis constant fixed, along with *one* of its component rate constants, while varying the two remaining rate constants. In figure 8*a*, we hold *K*_1_ and *k*_1_ constant, while increasing *d*_1_ and *a*_1_ in a proportion that maintains the constant *K*_1_. The profiles that are generated with these changes are all identical. By contrast, when we alter *k*_1_ and *a*_1_ (figure 8*b*) and *k*_1_ and *d*_1_ (figure 8*c*) we observe clear differences amongst the profiles. We also observe that across figure 8*b*,*c*, dose–response profiles associated with the matching values of *K*_1_ and *k*_1_ are the same irrespective of the values of *a*_1_ and *d*_1_. These same behaviours can be observed in the second (figure 8*d*–*f* ) and third (figure 8*g*–*i*) reactions.

**Figure 9.**
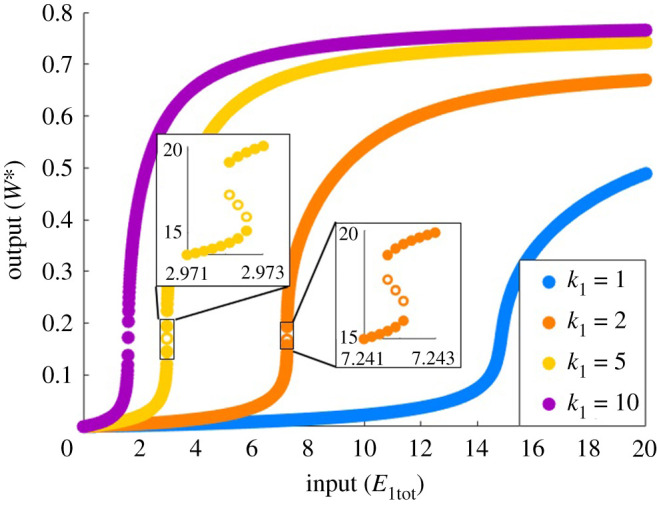
The role of the catalytic constant *k*_1_ in PAR-d. Parameters: $a_{1}=d_{1}=a_{2}=d_{2}=k_{2}=d_{3}=k_{3}=1$, $a_{3}=0.01$, $W_{\text{tot}}=100$, $E_{2\text{tot}}=20$. Closed circles indicate stable steady states; open circles are unstable steady states. (Online version in colour.)

In fact, it is clear that the catalytic constants (*k*_*i*_) play a unique and important role in tuning the shape of the system’s dose–response profiles. In figures 7*a*–*d* and 8, the parameters are altered to increase the catalytic constant in question without also altering the corresponding Michaelis constant. On the other hand, by increasing *k*_1_ on its own, and hence increasing *K*_1_ simultaneously, the system changes from monostable ultrasensitivity, to bistability, and then back to monostable ultrasensitivity (figure 9), an unusual and unexpected behaviour not afforded by the simple Michaelian model. Remarkably, the monostable regions of these profiles continue to increase in sensitivity, as this complex phenomenon unfolds, despite the inclusion of the bistable region. This behaviour is tied uniquely and specifically to the first catalytic constant, *k*_1_.

**Figure 10.**
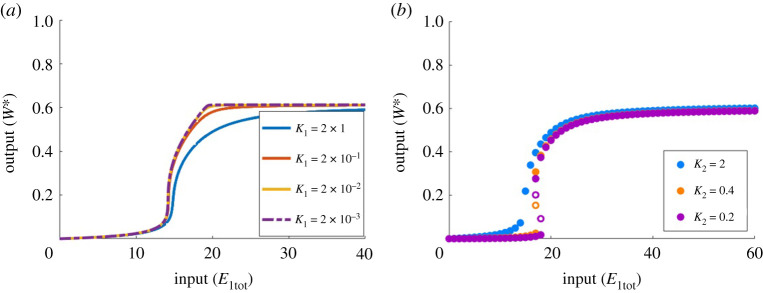
The role of Michaelis constants *K*_1_ and *K*_2_ in PAR-d. (*a*) *K*_1_ is decreased by increasing the value of *a*_1_ while holding *d*_1_ and *k*_1_ fixed. Parameters: $W_{\text{tot}}=100$, $E_{2\text{tot}}=20$, $d_{1}=k_{1}=a_{2}=d_{2}=k_{2}=d_{3}=k_{3}=1$; $a_{3}=1$ (blue), $a_{1}=10$ (red), $a_{1}=100$ (yellow) and $a_{1}=1000$ (purple). (*b*) *K*_2_ is decreased by increasing the value of *a*_2_ while holding *d*_2_ and *k*_2_ fixed. Parameters: $W_{\text{tot}}=100$, $E_{2\text{tot}}=20$, $a_{1}=d_{1}=k_{1}=d_{2}=k_{2}=d_{3}=k_{3}=1$, $a_{3}=0.01$; $a_{2}=1$ (blue), $a_{2}=5$ (orange) and $a_{2}=10$ (purple). Solid circles indicate stable steady states; open circles indicate unstable steady states. (Online version in colour.)

Moreover, while PAR-d can exhibit ultrasensitivity, as demonstrated in figures 8 and 9, the steepness of this near-vertical region of the dose–response cannot be increased indefinitely, in marked contradistinction to the Michaelian model, which supports unlimited ultrasensitivity. As shown in figure 9, increasing *k*_1_ can increase the sensitivity of the dose–response profile, but the steepness that can be achieved is limited. In addition, the location of the ultrasensitive portion of the curve is consistently moved to the left, towards the origin, which also constrains the development of ultrasensitivity due to this particular parameter change. In figure 10*a*, decreasing *K*_1_ with fixed *k*_1_ highlights the limit in ultrasensitivity that is approached as *K*_1_ is reduced.

Altering the other parameters of the PAR-d model (*K*_2_, *K*_3_ and *W*_tot_), in an attempt to increase sensitivity, ultimately culminates in the onset of bistability. Unlike the situation illustrated in figure 9, where the system switches from monostable to bistable and back to monostable for a monotone increase in a single parameter, bistability now persists once it is triggered at a critical value of the parameter in question. In particular, in figure 10*b*, we observe that decreasing *K*_2_ increases the sensitivity in the lower half of the substrate profile, without greatly affecting the sensitivity in the upper half. This ultimately leads to bistability as the lower half is shifted too far to the right. Further decreases in *K*_2_ do not lead to the creation of a one-way switch, as we illustrate by the profiles for $K_{2}=0.4$ and $K_{2}=0.2$, which can be observed to overlap without significant change.

**Figure 11.**
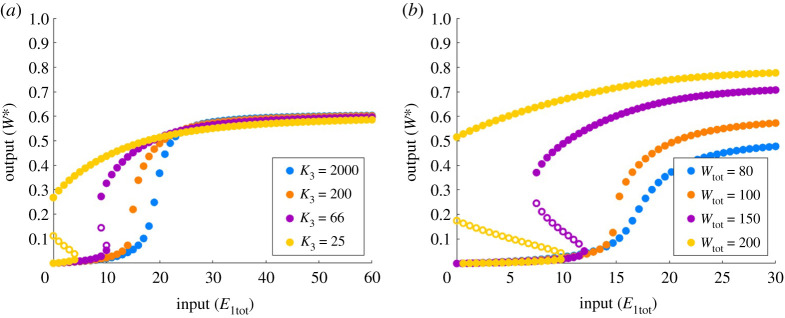
The role of Michaelis constant, *K*_3_, and protein abundance, *W*_tot_, in PAR-d. Parameters: $E_{2\text{tot}}=20$, $a_{1}=d_{1}=k_{1}=a_{2}=d_{2}=k_{2}=d_{3}=k_{3}=1$. (*a*) $W_{\text{tot}}=100$, with $a_{3}=0.001$ (blue), $a_{3}=0.01$ (orange), $a_{3}=0.03$ (purple) and $a_{3}=0.08$ (yellow). (*b*) $a_{3}=0.01$, with $W_{\text{tot}}=80$ (blue), $W_{\text{tot}}=100$ (orange), $W_{\text{tot}}=150$ (purple) and $W_{\text{tot}}=200$ (yellow). Solid circles indicate stable steady states; open circles indicate unstable steady states. (Online version in colour.)

For the parameters *K*_3_ (figure 11*a*) and *W*_tot_ (figure 11*b*), on the other hand, continued monotone alterations in these parameters transform the two-way switch into a one-way switch. By either decreasing *K*_3_ or increasing *W*_tot_, the upper portion of the dose–response profile is shifted rapidly to the left, much more rapidly than the shifting of the lower portion, which leads to an overlap, creating bistability. This trend continues and ultimately leads to the creation of a one-way switch as the upper portion of the curve moves to the left past the location of the vertical axis.

### (b) PAR-i exhibits ultrasensitivity and two-way (*not* one-way) switches, and admits a ‘prozone’ effect

Unlike the Michaelian model, wherein the two Michaelis constants are the drivers of bistability, or the PAR-d model, where a number of parameters drive bistability as discussed in the previous section, our extensive numerical simulations reveal that the catalytic constant *k*_4_ is the *only* parameter in the PAR-i model that drives bistability (figure 7*e*–*h*).

**Figure 12.**
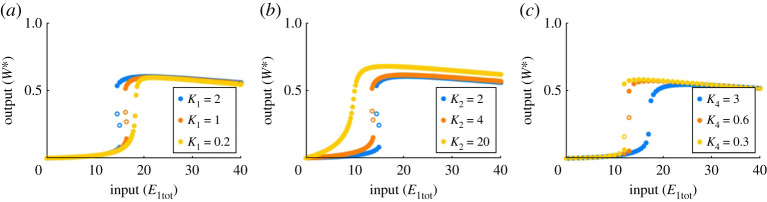
Michaelis constants can predispose PAR-i to bistability. Parameters: $W_{\text{tot}}=100$, $E_{2\text{tot}}=20$, $a_{1}=d_{1}=k_{1}=a_{2}=d_{2}=k_{2}=a_{4}=d_{4}=1$, $a_{3}=0.1$, $d_{3}=10$. (*a*) $k_{4}=5$, $a_{1}=1$ (blue), $a_{1}=2$ (orange) and $a_{1}=10$ (yellow); (*b*) $k_{4}=5$, $a_{2}=1$ (blue), $a_{2}=0.5$ (orange) and $a_{2}=0.1$ (yellow); (*c*) $k_{4}=2$, $a_{4}=1$ (blue), $a_{4}=5$ (orange), $a_{4}=10$ (yellow). Solid circles indicate stable solutions; empty circles indicate unstable solutions. (Online version in colour.)

The relationship between bistability and the parameter regime in the PAR-i model is quite subtle. In particular, while increasing the value of *k*_4_ is able to convert a sensitive but monostable profile into a bistable one, it is striking to observe that many other parameters can *predispose* the system to bistability, enabling the bifurcation to occur at a lower value of *k*_4_. In figure 12, for instance, we show that for a given value of *k*_4_, we are able to switch between ultrasensitivity and bistability by increasing the sensitivity of the system via a reduction in the Michaelis constants. In particular, decreasing the values of *K*_2_ and *K*_4_ increases the sensitivity of the underlying ultrasensitive monostable profile, allowing bistability to be triggered for smaller values of *k*_4_. Interestingly, when *K*_1_ is decreased, the system is ‘pushed back’ towards monostability. Nevertheless, Michaelis constants are not considered drivers of bistability in the sense that, for sufficiently low values of *k*_4_, they are unable to achieve bistability on their own.

**Figure 13.**
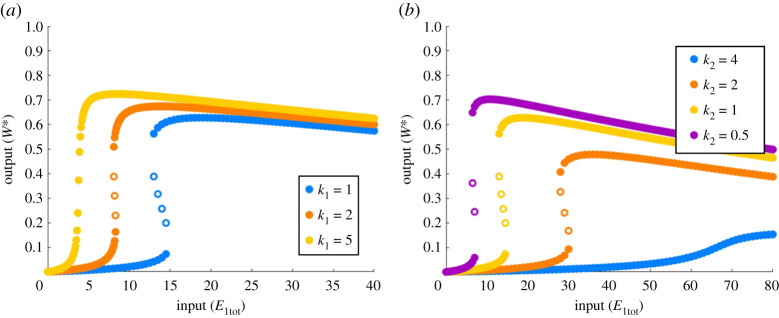
Low catalytic constants predispose PAR-i to bistability. Parameters: $W_{\text{tot}}=100$, $E_{2\text{tot}}=20$, $a_{1}=d_{1}=k_{1}=a_{2}=d_{2}=k_{2}=a_{4}=d_{4}=1$, $a_{3}=0.1$, $d_{3}=10$, and $k_{4}=10$. (*a*) $a_{1}=d_{1}=k_{1}=1$ (blue), $a_{1}=d_{1}=k_{1}=2$ (orange), $a_{1}=d_{1}=k_{1}=5$ (yellow); (*b*) $a_{2}=d_{2}=k_{2}=4$ (blue), $a_{2}=d_{2}=k_{2}=2$ (orange), $a_{2}=d_{2}=k_{2}=1$ (yellow), $a_{2}=d_{2}=k_{2}=0.5$ (purple). Solid circles indicate stable steady states; open circles indicate unstable steady states. (Online version in colour.)

In common with the behaviour of PAR-d, we found that the catalytic constant, *k*_*i*_, is the only component rate constant in each Michaelis constant, *K*_*i*_, that exerts a qualitative influence on the shape of the system’s dose–response profile. Moreover, in figure 13*b*, for instance, we see that decreasing *k*_2_ increases the sensitivity of the profile, and may thereby predispose the system to bistability. Increasing the total abundance of protein substrate, *W*_tot_, similarly increases the sensitivity of the profile, and may likewise predispose the system to bistability (not shown). On the other hand, increasing *k*_1_ increases sensitivity while also promoting monostability (figure 13*a*). In fact, we consistently found throughout our simulations that there is a very strong relationship between the values of *k*_1_ and *k*_4_, and the shape of the dose–response curve. In particular, for $k_{1}=k_{4}$, this system is unable to achieve bistability, regardless of how the other parameters are altered.

Unlike the PAR-d model, but in common with the Michaelian model, PAR-i can only exhibit two-way (reversible) bistable switches, not one-way switches. As we showed in figure 7*e*–*h*, continued increases in the key parameter *k*_4_ do not culminate in a one-way switch. This is to be expected, of course, because when $E_{1\text{tot}}=0$ for this particular PAR mechanism, there is no possibility of a ‘forward’ reaction in which W* is created, and thus no opportunity for a non-zero steady state in W*.

**Figure 14.**
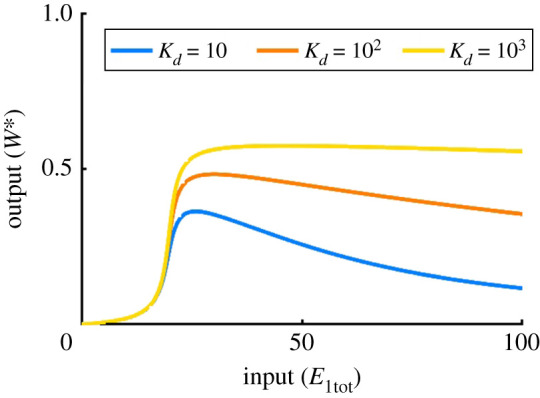
The dissociation constant in PAR-i and its ‘prozone’ effect. Parameters: $W_{\text{tot}}=100$, $E_{2\text{tot}}=20$, $a_{1}=d_{1}=k_{1}=a_{2}=d_{2}=k_{2}=a_{4}=d_{4}=k_{4}=1$, $d_{3}=10$; $a_{3}=1$ (blue), $a_{3}=0.1$ (red), $a_{3}=0.01$ (yellow). (Online version in colour.)

A further notable distinction between PAR-i and both the Michaelian and PAR-d models is the eventual decrease in system output with continued increases in input (*E*_1tot_), effectively a type of ‘prozone’ effect [85]. The prozone effect was originally observed in the context of immunological reactions, but has been observed more recently in cellular signalling networks, most notably in the processing of biochemical signals via scaffold proteins [85]. By analysing the abundances of intermediate complexes in this scenario, we find that after the maximum conversion from *W* to W* is achieved (peak in W* profile), and *E*_1tot_ continues to be increased, the third complex, *C*_3_, continues to be produced without being consumed in the fourth reaction (due to insufficient *W*). This phenomenon is unique to the PAR-i model, and can be controlled by altering the value of $K_{d}=d_{3}/a_{3}$. Indeed, we find that by increasing *K*_*d*_, we can mitigate this loss in maximum output (figure 14). Of course, inhibiting the formation of this complex in this manner also reduces the PAR contribution to the covalent-modification cycle.

### (c) Unlimited ultrasensitivity is difficult to achieve in complex-complete PAR models

**Figure 15.**
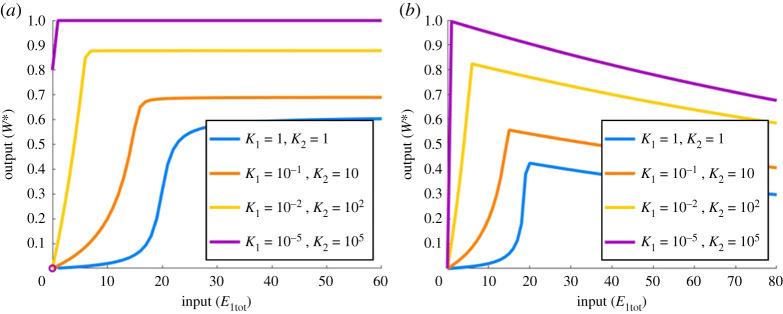
Parameter conditions corresponding to unlimited ultrasensitivity in PAR-MM, applied to PAR-d and PAR-i. In PAR-MM, unlimited ultrasensitivity obtains in the limit as $K_{1}\to 0$ and $K_{2}\to \infty$ (i.e. $K_{1}\ll W_{\text{tot}}-W^{*}$ and $K_{2}\gg W^{*}$). (*a*) PAR-d and (*b*) PAR-i, under these same parametric conditions. Parameters: $W_{\text{tot}}=100$, $E_{2\text{tot}}=20$, $d_{1}=k_{1}=d_{2}=k_{2}=1$; $a_{1}=1$, $a_{2}=1$ (blue); $a_{1}=10^{-1}$, $a_{2}=10$ (orange); $a_{1}=10^{-2}$, $a_{2}=10^{2}$ (yellow); $a_{1}=10^{-5}$, $a_{2}=10^{5}$ (purple). (*a*) $a_{3}=10^{5}$, $d_{3}=k_{3}=1$, $K_{3}=10^{-5}$; (*b*) $a_{3}=0.1$, $d_{3}=10$, $a_{4}=10^{5}$, $d_{4}=k_{4}=1$, $K_{d}=100$, $K_{4}=10^{-5}$. (Online version in colour.)

Although ultrasensitive profiles could be obtained for both the PAR-d and the PAR-i models, it is important to consider whether or not the models support *unlimited* ultrasensitivity. In other words, can the slope of the highly sensitive (near-vertical) region of the dose–response profile be made *arbitrarily steep* in some parameter limit? Unlimited ultrasensitivity is of central importance to the implementation of RPA, and was incorporated into the influential RPA study by Ma *et al.* [13] using the Michaelian model (PAR-MM) discussed in the present work. Because PAR-MM is capable of achieving unlimited ultrasensitivity in the parameter limit $K_{1}\to 0$ and $K_{2}\to \infty$ ($K_{1}\ll W_{\text{tot}}-W^{*}$ and $K_{2}\gg W^{*}$), we examined the performance of our PAR-d and PAR-i models in this specific parameter regime. As we illustrate in figure 15, we are unable to achieve unlimited ultrasensitivity using these conditions for any choice of the remaining parameters in the model. In fact, for both the PAR-d and PAR-i models, as the limits $K_{1}\to 0$ and $K_{2}\to \infty$ are approached, the system exhibits a steep linear increase in W* with *E*_1tot_ close to the origin.

**Figure 16.**
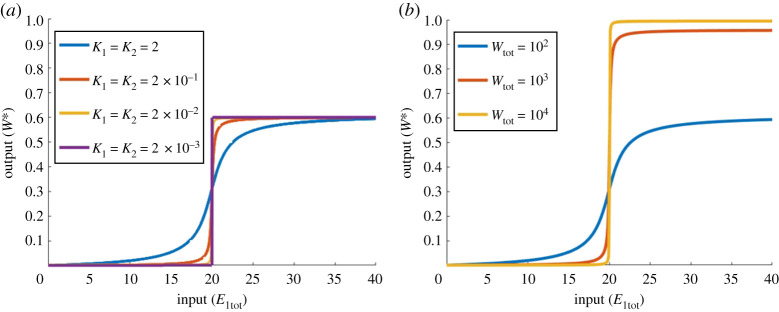
Unlimited ultrasensitivity in PAR-d parameters: $W_{\text{tot}}=100$, $E_{2\text{tot}}=20$, $a_{1}=d_{1}=k_{1}=a_{2}=d_{2}=k_{2}=d_{3}=k_{3}=1$, $a_{3}=10^{-10}$. (*a*) $a_{1}=a_{2}=1$ (blue), $a_{1}=a_{2}=10$ (red), $a_{1}=a_{2}=10^{2}$ (yellow); $a_{1}=a_{2}=10^{4}$ (purple); (*b*) $W_{\text{tot}}=10^{2}$ (blue), $W_{\text{tot}}=10^{3}$ (red), $W_{\text{tot}}=10^{4}$ (yellow). Almost identical profiles can be achieved in PAR-i by setting $a_{3}=10^{-10}$, $d_{3}=10$ and $a_{4}=d_{4}=k_{4}=1$ to remove the effect of PAR (not shown). (Online version in colour.)

Our extensive numerical simulations revealed that the *only* parametric condition that yields unlimited ultrasensitivity in the PAR-d model is $a_{3}\to 0$ ($K_{3}\to \infty$), along with either $K_{1}\to 0$ and $K_{2}\to 0$ (figure 16*a*) or $W_{\text{tot}}\gg E_{2\text{tot}},E_{1\text{tot}}$ (figure 16*b*). We note that the first of these conditions ($a_{3}\to 0$) corresponds to the limit in which the PAR-d mechanism approaches the Goldbeter–Koshland model [46], i.e. a simple covalent-modification cycle *without* PAR. In addition, the second set of conditions ($K_{1}\to 0$ and $K_{2}\to 0$ or $W_{\text{tot}}\gg E_{2\text{tot}},E_{1\text{tot}}$) corresponds to the parameter regime in which Goldbeter & Koshland [46] achieved unlimited ultrasensitivity via the zero-order mechanism. In other words, PAR-d can only exhibit unlimited ultrasensitivity once the PAR contribution is removed. PAR-d, in its essence, appears to be incapable of unlimited ultrasensitivity.

**Figure 17.**
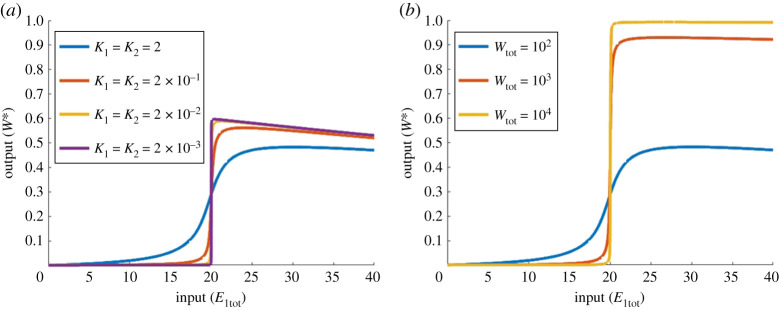
Unlimited ultrasensitivity in PAR-i. Parameters: (both plots) $E_{2\text{tot}}=20$, $d_{1}=k_{1}=d_{2}=k_{2}=a_{4}=d_{4}=k_{4}=1$, $a_{3}=0.1$, $d_{3}=10$; (*a*) $W_{\text{tot}}=100$; $a_{1}=a_{2}=1$ (blue), $a_{1}=a_{2}=10$ (red), $a_{1}=a_{2}=10^{2}$ (yellow) and $a_{1}=a_{2}=10^{3}$ (purple); (*b*) *a*_1_ = $a_{2}=1$; $W_{\text{tot}}=10^{2}$ (blue), $W_{\text{tot}}=10^{3}$ (red), $W_{\text{tot}}=10^{4}$ (yellow). (Online version in colour.)

As for our PAR-i model, unlimited ultrasensivity was possible under very specific conditions. First, in common with PAR-d, unlimited ultrasensitivity may be realized in the parameter limit by which the Goldbeter–Koshland model [46] is recovered from the PAR-i model. This gives rise to dose–response profiles that are identical to figure 16. But in addition to this, PAR-i is also able to engender unlimited ultrasensitivity in an additional set of conditions, where PAR remains present in the model. Specifically, we consistently found that provided $k_{1}\approx k_{2}\approx k_{4}$, unlimited ultrasensitivity may arise in the limit as $K_{1}\to 0$ and $K_{2}\to 0$ (figure 17*a*), and/or in the limit as $W_{\text{tot}}\to \infty$ (figure 17*b*).

Thus, unlimited ultrasensitivity is difficult to achieve in our complex-complete models of PAR, in the sense that it requires that either (i) the positive autoregulatory mechanism be removed completely, through the vanishing of a relevant parameter; or (ii) in the case of PAR-i, a very specific parameter selection ($k_{1}\approx k_{2}\approx k_{4}$) be in place, in addition to other ultrasensitivity-promoting parameter limits. Given that biochemical systems are generally unable to exercise control over the numerical values of their parameters, we therefore do not expect unlimited ultrasensitivity to be achievable in practice for either complex-complete PAR mechanism.

## 3. Discussion and concluding remarks

The Michaelis–Menten equation, and variations thereon, has appeared almost ubiquitously in mathematical descriptions of enzyme-mediated chemical reactions for more than 100 years [81,82], often quite indiscriminately, with little or no consideration of whether it is able to truly recapitulate the behaviour of the (potentially much more complicated) system being modelled. In particular, it has been used in highly influential work [13] to suggest that PAR of a covalent-modification cycle can generate unlimited ultrasensitivity, and as a consequence, RPA when suitably embedded in certain network topologies. But the mathematical consequences of approximating complex and intricate molecular interactions (that could be present in added regulations such as PAR) by a Michaelian rate law have not previously been rigorously examined. Here we ask: Can a Michaelian description of such a system accurately represent the detailed molecular mechanisms that must be present in a ‘real’ collection of interacting molecules, which could, in principle, be quite intricate and complicated? And can it truly recapitulate the range of qualitative behaviours afforded by more detailed mass-action descriptions that account for all molecular interactions? Our answer, based on two detailed mass-action models that carefully account for the specific molecular mechanisms through which PAR is actually transacted, and explicitly account for all intermediate molecular species—a framework we call ‘complex-complete’—is a resounding *no*.

As noted elsewhere in this work, ultrasensitivity can contribute to the implementation of RPA because the near-vertical region of the ultrasensitive dose–response can be transformed to a near-horizontal (invariant) response to an external network stimulus, once the ultrasensitivity-generating mechanism is embedded into a negative feedback loop [14,15]. Of course, in practice, ‘real’ biological systems may tolerate some imprecision in the adaptation mechanism, such that *some* degree of embedded ultrasensitivity may be adequate. Nevertheless, the capacity of a collection of embedded reactions to exhibit *unlimited ultrasensitivity* is functionally significant: unlimited ultrasensitivity corresponds to the potential to ‘tune’ the steepness of the dose–response, either through alterations to gene expression (protein abundance, such as *W*_tot_ in this case) or through alterations in rate constants via mutation, to be as steep as needed for the requisite adaptation response. Both the Goldbeter–Koshland model [46] and the Michaelian model of PAR proposed by Ma *et al*. [13] (here, PAR-MM) are capable of unlimited ultrasensitivity in this sense.

By contrast, our two complex-complete models of PAR—in which we consider the specific mechanism by which PAR may be encoded, and explicitly incorporate all molecular details of the mechanism into the mathematical model—exhibit limitations in the steepness of the slope in the ultrasensitive region of the dose–response. In particular, parameter alterations that are conducive to increased ultrasensitivity ultimately trigger the onset of bistability. In this sense, the ultrasensitivity engendered by these more detailed models is far more fragile than is predicted by the simplified Michaelian approximation (PAR-MM). In the context of RPA, then, whereas simplified (Michaelian) models may predict that parameter perturbations simply reduce the precision of adaptation (which may be functionally acceptable to the biological system as a whole), our more detailed analysis suggests that the capacity for adaptation could actually be lost altogether.

On the other hand, both our complex-complete models were capable of bistability, a qualitative response that is also realized by the Michaelian simplification (PAR-MM). But whereas both the PAR-MM model and our PAR-i model could only exhibit two-way (reversible) bistable switches, our PAR-d model could also exhibit one-way (irreversible) switches. Clearly mechanism matters: not only is the Michaelian model unable to fully recapitulate the qualitative responses of the more detailed complex-complete models, but also the choice of specific PAR mechanism encoded by the complex-complete framework also plays an important role. While the distinction between one-way and two-way switches may be functionally unimportant for biological systems that predominantly operate in a regime far removed from the thresholds at which bistable switches are ‘tripped’, bistable switches are thought to play a central role in signalling phenomena such as apoptosis and motility signalling [4,5], among others. In this context, the existence of a one-way switch, rather than the two-way switch suggested by Michaelian and PAR-i models of PAR, could have profound consequences, particularly in the development of therapeutic strategies. In particular, a pharmacological agent that ablates the incoming signal to a one-way switch will not be able to modulate downstream signalling once the switch has been ‘tripped’.

Thus, our study adds to a growing body of literature that suggests that more caution should be exercised in the use of the Michaelis–Menten equation to model enzyme-mediated reactions. Our study particularly emphasizes that the use of the Michaelis–Menten equation may be inadequate for the functional elucidation of complex signalling networks, especially when additional regulations (such as autoregulation) are present. This is particularly important in our current ‘big data’ era, as the systems biology community moves increasingly in the direction of large-scale network models and ‘whole-cell modelling’ [86]. The sheer size and complexity of these models, and the need to reconcile theory with experimental data, naturally introduce enormous challenges in terms of model parameterization and parameter inference. While it is expedient in this context to make mathematical representations of the system as simple as possible, our study underscores the potential dangers of doing so.

We note in closing that complex-complete mass-action models, as we propose here, constitute polynomial dynamical systems whose steady-state solutions form algebraic varieties. Significant progress has been made in recent years in the application of Gröbner basis methods to the study of these kinds of models (e.g. [87]). Nevertheless, because Gröbner bases can quickly become large and expensive to compute as the underlying models become more complicated, numerical simulations may still be required in many cases to support the analysis of mechanism-based models of complex networks.

## 4. Methods

Numerical simulations and root-finding methods were all undertaken using standard packages available in Matlab (ODE45, ODE23s, roots and fsolve). Total number of possible steady states in the interval [0,*W*_tot_] were checked by Gröbner basis calculations (and subsequent numerical examination in Matlab) available in the open-source package Singular (https://www.singular.uni-kl.de/).

## Data accessibility

All code available from the authors upon reasonable request. All code used for this article is available via GitHub: https://github.com/JeynesSmith/JeynesSmithUltrasensitivity2021.
